# Multichannel optrodes for photonic stimulation

**DOI:** 10.1117/1.NPh.5.4.045002

**Published:** 2018-10-23

**Authors:** Yingyue Xu, Nan Xia, Michelle Lim, Xiaodong Tan, Minh Ha Tran, Erin Boulger, Fei Peng, Hunter Young, Christoph Rau, Alexander Rack, Claus-Peter Richter

**Affiliations:** a Northwestern University Feinberg School of Medicine, Department of Otolaryngology, Chicago, Illinois, United States; b Northwestern University, Department of Communication Sciences and Disorders, Evanston, Illinois, United States; c Qingdao University, Institute for Digital Medicine and Computer-assisted Surgery, Qingdao, China; d Chongqing University, Bioengineering College, Chongqing, China; e Diamond Light Source Ltd., Diamond House, Harwell Science and Innovation Campus, Didcot, Oxfordshire, United Kingdom; f Structure of Materials Group-ID19, European Synchrotron Radiation Facility, Cedex 9, France; g Northwestern University, Department of Biomedical Engineering, Evanston, Illinois, United States; h Northwestern University, Hugh Knowles Center for Clinical and Basic Sciences in Hearing, Evanston, Illinois, United States

**Keywords:** infrared neural stimulation, light delivery system, laser, optrode, cochlear implant

## Abstract

An emerging method in the field of neural stimulation is the use of photons to activate neurons. The possible advantage of optical stimulation over electrical is attributable to its spatially selective activation of small neuron populations, which is promising in generating superior spatial resolution in neural interfaces. Two principal methods are explored for cochlear prostheses: direct stimulation of nerves with infrared light and optogenetics. This paper discusses basic requirements for developing a light delivery system (LDS) for the cochlea and provides examples for building such devices. The proposed device relies on small optical sources, which are assembled in an array to be inserted into the cochlea. The mechanical properties, the biocompatibility, and the efficacy of optrodes have been tested in animal models. The force required to insert optrodes into a model of the human scala tympani was comparable to insertion forces obtained for contemporary cochlear implant electrodes. Side-emitting diodes are powerful enough to evoke auditory responses in guinea pigs. Chronic implantation of the LDS did not elevate auditory brainstem responses over 26 weeks.

## 1 Introduction

### 1.1 Cochlear Implants and Their Challenges

Cochlear implants (CIs) are considered one of the most successful neural prostheses. Today about 350,000 individuals with severe-to-profound hearing loss have received a CI to restore some of their hearing. However, the performance of individual users varies largely. While some patients are able to communicate over the phone in different languages, others receive little benefit from CIs. For all CI users, noisy listening environments and music perception constitute a challenge.^1^^–^^3^ It has been argued that performance could be improved by reducing the interaction between neighboring CI electrode contacts, subsequently creating more independent channels for stimulation. More spatially selective stimulation with electric current can be achieved through multipolar stimulation, where multiple electrode contacts are used to narrow the current field.^4^^–^^7^ Another approach to increasing the number of different pitch percepts is called current steering.^8^^–^^10^ In this approach, neighboring electrodes are used simultaneously to “steer” the current to selected neuron populations between the two contacts. However, this technique does not introduce more independent channels for parallel stimulation.^10^^–^^14^

### 1.2 Optical Stimulation

More recently, the use of photons has been suggested as an approach to evoke responses from small populations of neurons,^15^^–^^18^ because optical radiation can be delivered spatially selectively.^19^^,^^20^ It is anticipated that optical stimulation decreases interactions between neighboring channels, allowing for the development of neural prostheses with enhanced neural fidelity. Two methods for direct neural stimulation with light are currently considered: optogenetics and infrared neural stimulation (INS).^15^^,^^16^^,^^18^^,^^21^ Optogenetics requires the delivery of a viral vector to express photosensitive ion channels in the membrane of the spiral ganglion neurons (SGNs).^16^^,^^22^ INS does not require such treatment because during INS, the fluid in the target tissue absorbs the photons and the energy is converted into heat.^23^^–^^27^ Spatially and temporally confined heating evokes action potentials in the SGNs (see below for mechanism). Although both methods appear promising, they also have challenges. For optogenetics, the neurons must be manipulated genetically. This requires targeting of a selected population of neurons with a viral vector to induce stable expression of light-sensitive ion channels. The rate by which the ion channel or optogenetic tool is expressed is crucial since low expression of the optogenetic tool will require larger photon flux rates and high expression of the ion channel may damage the cell. Moreover, tissue in the beam path largely scatters the incident photons resulting in broad response profiles and a significant reduction of the transmitted radiant energy. Published results have shown that the energy required to evoke an action potential on the murine auditory nerve is about 10-times larger than for electrical stimulation.^16^ The challenge for INS is the delivery of heat to the target structure(s), which needs to be removed or dissipated to prevent thermal damage during stimulation. Tissue heating limits the rate of stimulation to about 250 pulses per second (pps) at a maximal radiant energy of 25  μJ/pulse.^28^^–^^31^ Optical sources, small enough to be inserted into the cochlea, have a low-wall-plug efficiency (ratio in converting electrical power to optical radiation). The energy required to stimulate with INS is about 100 times larger when compared to electrical stimulation.^18^^,^^32^ Optoacoustic events resulting from INS must be considered for patients with residual hearing.

When comparing optogenetics and INS, the wavelengths of the radiation used for each of the two methods should also be considered. The incident radiant energy is reduced by the tissue in the beam path through scattering and absorption of the photons. At wavelengths used for optogenetics, λ<1064  nm, the extinction coefficient for the radiation is governed by the scattering of the photons, whereas above λ=1064  nm, the absorption becomes the dominant factor in tissue. Hence, for optogenetics, tissue reduces the transmitted radiant energy and broadens the beam by scattering. For INS, the energy is mostly reduced through absorption of the photons and less through scattering.^33^^,^^34^

### 1.3 INS-Neurons are Activated by Temporally and Spatially Confined Heating

One of the first reports on laser irradiation as a method to stimulate neurons came from Fork’s study on *Aplysia Californica*.^35^ Irradiation of the tissue with blue (λ=488  nm) light (spot size=10  μm) evoked action potentials at stimulus levels above 12.5 mW radiation power.^35^ Wells and coworkers studied light tissue interactions using the free-electron laser in great detail. They determined radiation wavelengths that could be used for safe neural stimulation, which are in the near-infrared and infrared.^36^ One of the wavelength ranges for which optical sources exist for stimulation is between 1840 and 2100 nm. Upon the absorption of the photons by the water, their energy is converted into heat,^23^ which then evokes an action potential. Temporally and spatially confined heating depolarizes the cell by changing the membrane capacitance^37^^–^^40^ resulting in a depolarizing inward current. The change in capacitance might result from changes in membrane thickness^41^ or from small-diameter nanopores in the membrane.^42^ Furthermore, it has been demonstrated that transient receptor potential cation channels of the vanilloid group (TRPV) are involved.^26^^,^^43^^–^^45^ They are temperature sensitive and are highly calcium selective.^46^^–^^55^ Published results demonstrated that intracellular calcium homeostasis changes during INS.^56^^–^^61^ Spatially and temporally confined heating, which occurs during INS, also results in stress relaxation waves.^62^ Those pressure waves could vibrate the basilar membrane and evoke auditory responses through stimulation of remaining inner hair cells. Results have been presented where cochlear INS did not evoke responses in deaf animals.^63^^–^^65^ Tan et al. concluded that INS in the cochlea only originates from the generation of a pressure wave. Those findings differ from reports that showed responses in deaf animals missing hair cells^15^^,^^66^ and in congenitally deaf mice.^67^ One of the deaf mouse models lacks the vesicular glutamate transporter-3 ($\mathrm{VGLUT}3^{-/-}$) and does not release glutamate at the inner hair cell afferent synapse.^67^^–^^70^ This mouse model shows no auditory response to sound stimuli but responds to INS, indicating direct interactions between INS and SGNs. Other gene manipulated mice, which show no auditory brainstem response (ABR) response to acoustical stimuli, but respond to INS,^67^ are the Atoh1-cre; ${\mathrm{Atoh}1}^{f/\mathrm{kineurog}1}$ mice.^71^^,^^72^

To use INS in a CI, photons must be delivered to selected sites along the cochlea. This can be achieved by inserting light delivery systems (LDSs) into scala tympani of the basal turn of a cochlea. LDSs can be arrays of optical sources, such as side-emitting laser diodes (SELDs) or vertical cavity surface emitting lasers (VCSELs), bundles of glass fibers, or bundles of polyimide waveguides. Low $H_{2}O$ containing glass fibers are not considered as LDS because they are too stiff and break easily if they are larger than 50-μm in diameter. Stiff optical fiber bundles will also damage the cochlear soft tissue structures during insertion into scala tympani and cannot be inserted at sufficient lengths.^73^ Polyimide waveguides are flexible and biocompatible. However, challenges remain in coupling the light sources and waveguides. Moreover, the size of the waveguides limits the number of individual optical stimulation sites along the spiral ganglion.^73^

This paper discusses how to build and test optrodes with small optical sources, which are powerful enough for INS.

## 2 Methods

### 2.1 Light Sources

**Fig. 1:**
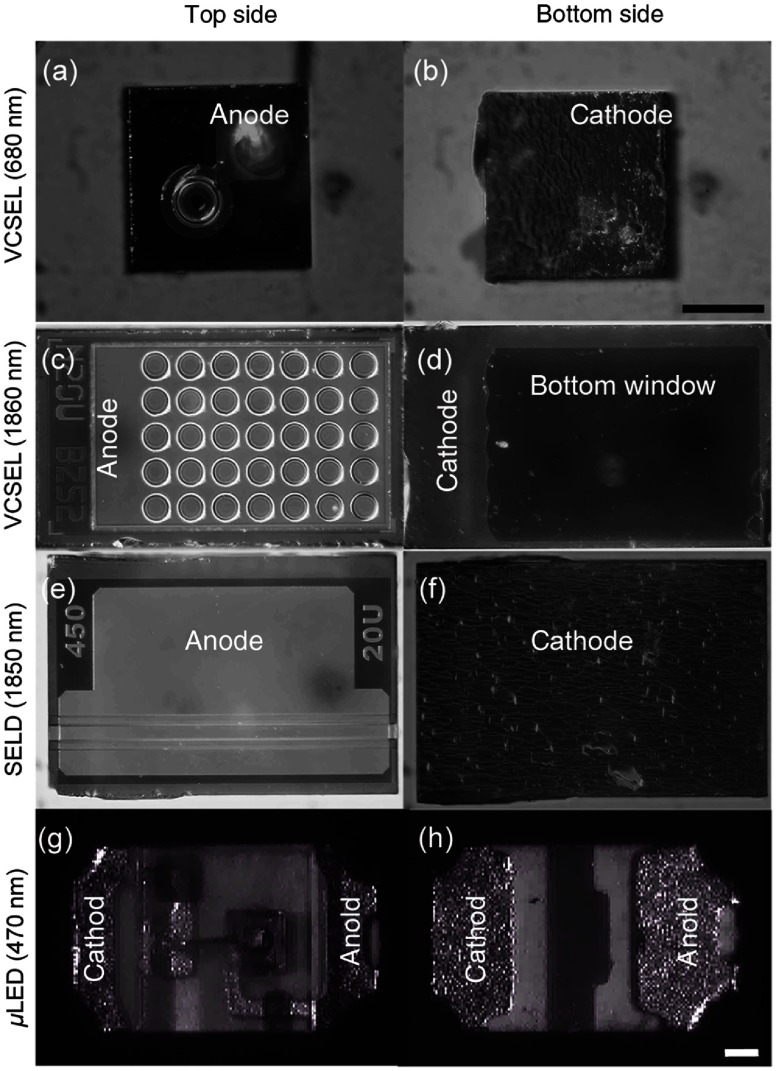
Different light sources. (a) The top and (b) the reverse side of a VCSEL (λ=680  nm). The dimension is $250\times 250\times 200\;\mu m^{3}$ and its maximum output power is about 4 mW. (c) and (d) The top and bottom of a 5×7 VCSEL array, (λ=1860  nm). The dimension is $450\times 250\times 200\;\mu m^{3}$ and its maximum output power is about 7.5 mW. Each circle in (c) represents one VCSEL. (d) The light emitting window and the cathode, (e) and (f) a SELD (λ=1850  nm). The dimension is $450\times 350\times 100\;\mu m^{3}$ and its maximum output power is about 50 mW. (g) The original appearance of a blue μLED (λ=470  nm) before being resized. The dimension is $1000\times 600\times 200\;\mu m^{3}$ and its maximum output power is about 34 mW. Scale bars are the same for (a)–(f) [shown in (b)] and (g)–(h) [shown in (h)]: 100  μm. The power ratings are given for continuous wavemode operation.

Currently, three different types of light sources have been used to fabricate optrodes, VCSELs, SELDs, and microlight-emitting diodes (μ-LEDs). The light sources are available in sizes that can be assembled into arrays suitable for insertion into scala tympani (Fig. 1). Considering the frequency place map in the human cochlea (organ of Corti and spiral ganglion),^74^^,^^75^ 50 light sources could be assembled into a 24-mm long array and provide a frequency resolution of about 1/8 of an octave.^74^^,^^75^ Our most recent arrays feature 15 light sources on a 24-mm long array. The number of optical sources will be increased in future optrodes by placing the optical dies closer together.

### 2.2 Optrodes and Hybrids

**Fig. 2:**
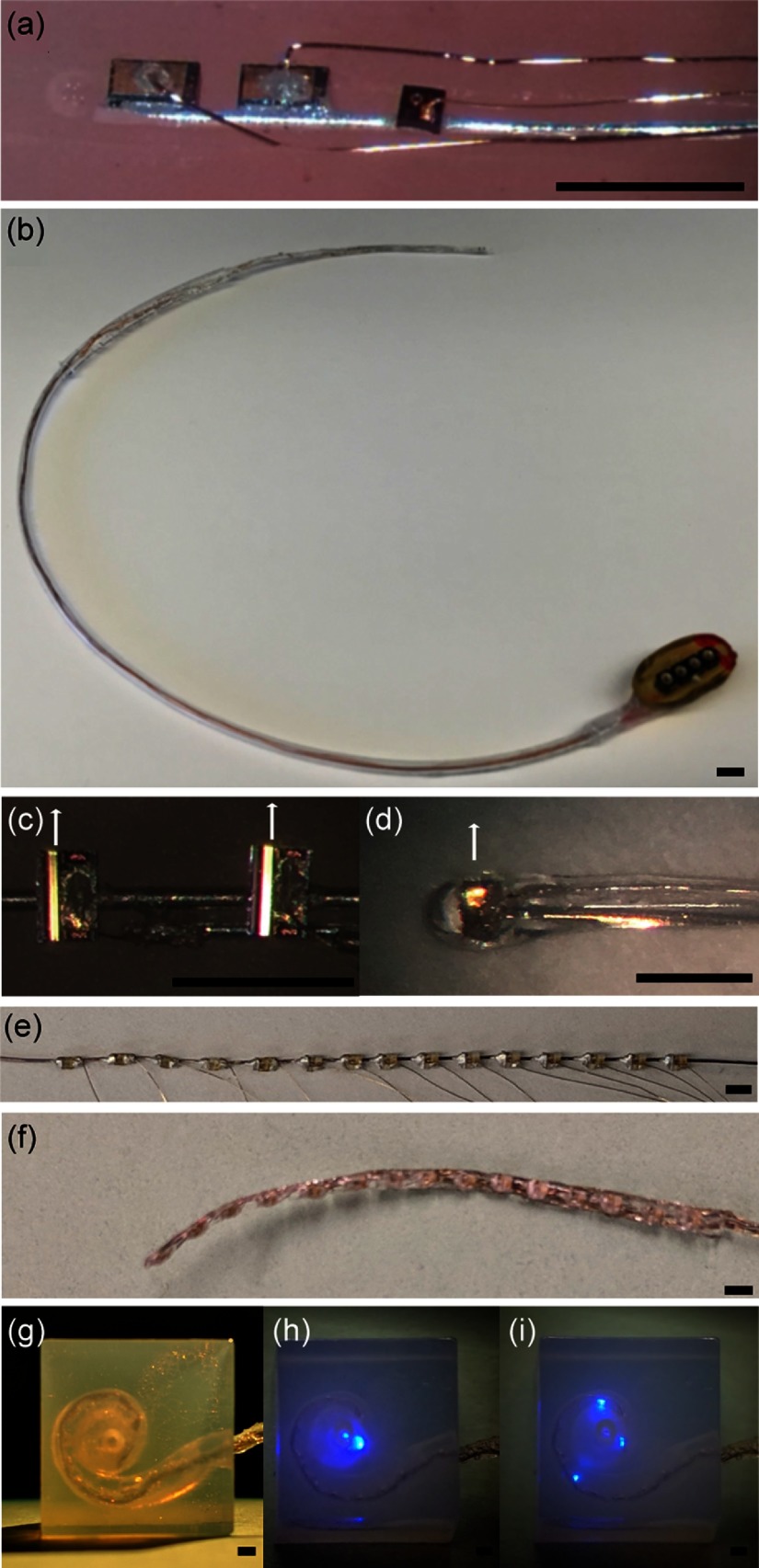
Optrodes fabricated with small optical sources. (a) A three-channel optrode. The two infrared sources in (a) are VCSELs (λ=1860  nm, larger dies to the left). A red source (λ=680  nm, smaller die to the right) serves as a pilot light, which helps to orient the implant. (b) The completed electrode ready for implantation. At the bottom right, the transcutaneous connector is shown. (c) A picture of a two-channel optical array made of SELDs. The size of the given emitters is 450  μm×300  μm×100  μm. The anode of each emitter was connected with a thin gold wire, and then to a Teflon-insulated silver wire. The cathodes of both emitters were connected to a single silver wire, which also served as a heat sink for the light sources. The direction of the light emission is indicated by the arrow. (d) A picture of a silicone-embedded single-channel optical array made with a SELD. The anode and cathode connections are the same as shown in panel (c). The array is embedded in silicone. (e) An array of 15  μLEDs connected with a silver wire to the cathodes and platinum wires to the anodes. (f) An optrode made with 15 light sources. (g) The insertion of this optrode into a human scala tympani model. (g) and (i) The radiation of a single μLED and multiple μLEDs. Scale bars=900  μm.

The first step in the fabrication of the multichannel optrode was connecting the cathode of the light sources with conductive silver epoxy (EPO-TEK H20E, Epoxy Technology Inc., Billerica, Massachusetts) to a 125-μm diameter silver wire [Figs. 2(a), 2(c), and 2(e)]. The distance between the sources could be adjusted as needed. The silver wire, which could be replaced by strips of platinum or silver foil, also serves as a heat sink. In the second step, a 25-μm diameter Teflon-coated platinum wire was connected to the anode of each source using conductive silver epoxy. Note that wire-bonding has been tested as an alternate contacting method [Fig. 1(a)], however, the connection is fragile and using epoxy resulted in more reliable connections of the light sources. Following the assembly of the optrode, the function of each light source was tested before and after the optrode was embedded into silicone. For the silicone embedding, the optrode was placed in a custom fabricated mold. The mold was filled with Silastic [MDX4-4210, Medical Grade Elastomere, base and curing agent (LOT 0006932899, Dow Corning Corp., USA)] and was allowed to cure overnight in an oven at 60°C. After the silicone was solidified, the electrode was removed from the mold [Figs. 2(b), 2(d), and 2(f)] and the wires of the optrode were extended to about 10 cm. Then the optrode could be inserted into a Tygon^®^ Micromix flexible microbore plastic tubing with an inner diameter of 1 mm and an outer diameter of 1.8 mm (LOT 507206, Saint-Gobain Performance Plastics, Portage, Wisconsin) and was connected to a transcutaneous connector [Fig. 2(b)]. During implantation, the transcutaneous connector was secured with Ethilon 3.0 (Ethicon, Cincinnati, Ohio) to the skin incision [Fig. 2(b)].

A different optrode design used Flexible Printed Circuit Board (FPCB) technology. The single-layer FPCB was designed as the light source carrier, which renders the optrode fabrication process much easier. The substrate for the CI must be soft, flexible, and biocompatible. It has been demonstrated that polyimide polymers are suitable in stiffness (see also discussion), are biocompatible,^76^^,^^77^^–^^80^ and were selected for the support base and the insulation cover layer. Copper was selected as the conductive material. Adhesive films provide the material to bond the copper foil to the base film. The epoxy and copper contacts were further coated with silicone for biocompatibility.

**Fig. 3:**
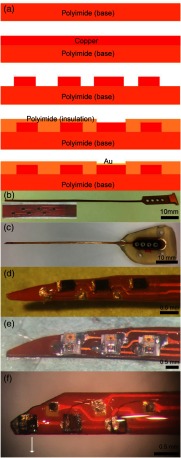
The fabrication of the optrode based on FPCB technique. (a) The vertical fabrication structure and materials of the light source carrier and (b) the FPCB carrier and the tip. The light source mounting area on the tip is 100  μm×100  μm; the distance between two channels is 1 mm; the width of the carrier is 0.75 mm, and the thickness is 100  μm. (c) An FPCB-based optrode with a connector at the end and (d) the tip of an FPCB-based optrode with three VCSELs. (e) The tip of a FPCB-based optrode with three μLEDs. (f) The tip of a FPCB-based optrode with three infrared SELDs.

To fabricate the multichannel optrode carrier, a 25-μm-thick copper foil was laminated on the upper surface of the polyimide substrate. Unwanted copper was etched from the copper layer, such that the resulting wire width was 80  μm. To isolate each channel, a 25  μm-thick polyimide film was laminated on the surface. Subsequently, the polyimide film applied for insulation was etched away on the top of the light source mounting areas and solder joints. Light source mounting areas and solder joints were further improved by electroplating a 25-μm-thick gold layer on the contact areas [Fig. 3(a)]. Dictated by the number of current sources of our portable diode driver system, we only fabricated three channel optrodes. The number of contacts, however, can easily be expanded. Figure 3(b) shows the FPCB carrier and its tip. Figure 3(c) shows the FPCB-based optrode with a connector. Figures 3(d)–3(f) show the FPCB carrier and a three-channel optrode made with VCSELs, μLEDs, and SELDs. This carrier can also accommodate other light sources and metal contacts for electrical stimulation.

### 2.3 Insertion Force Measurements

The insertion force of the electrodes and optrodes was measured in five cadaveric cat and human cochleae, as well as in a matrix printed model of the human scala tympani (∼0.016-mm resolution, Objet 260vs Dental, Fisher Unitech Corporation, Chicago, Illinois). The human cochleae were obtained through the Anatomy Gifts Registry. Each cochlea was accessed via a retro-facial approach and a cochleostomy was created 0.5 to 1 mm from the round window. This allowed the insertion of five short custom fabricated optrodes (with <5 contacts, Table 1). Four long arrays (with 10+ contacts, Table 1) were tested in the human scala tympani model. For the experiments, the electrodes/optrodes were either mounted on a Narishige electrical step-motor (MM108; Narishige, Japan) or an LTS150 translation stage (Thorlabs, Newton, New Jersey) to advance the optrode with constant speed at 0.553 or 0.25 mm/s, respectively. At the same time, the insertion force was measured with a Mark-10 Digital Force Gauge (Model M5-012, Mark-10 Corporation, Copiague, New York). The MESUR Lite by Mark-10 software was used to acquire the data at a sampling rate of 10 Hz. The electrode advancement was always monitored through a microscope and was stopped immediately when the electrode would not advance.

**Table 1:** The different arrays used for the insertion force measurements in a cat cochlea, human cochleae, and a human scala tympani model. The number of optical or electrical sources, the width, the height, insertion force, and distance are also listed. The four arrays equal or longer than 15 mm decrease their width and height from base to apex. Changes are given by the ranges.

Type	Cochlea	Contacts no.	Length (μm)	Width (μm)	Height (μm)	Insertion force (mN)	Insertion distance (μm)
μLED (blue)	Cat, human	4	5000	920	710	<5	2686
μLED (blue)	Cat, human	4	5000	790	620	<5	2965
μLED (blue) VCSEL (red)	Cat, human	5	6000	790	570	<5	3286
VCSEL (infrared)	Cat, human	3	4000	850	440	<5	2926
Electric	Cat, human	3	4000	780	540	<0.5	3552
μLED (blue)	Human model	10	15,000	1100 to 1000	900 to 600	<150	8275
μLED (blue)	Human model	15	24,000	1100 to 600	900 to 500	<130	13,230
Electric (commercial)	Human model	12	26,400	1100 to 600	1100 to 600	<75	16,530
Electric (commercial)	Human model	16	24,500	1100 to 600	600 to 400	<130	13,600

### 2.4 Testing in an Animal Model

All animal procedures were carried out in accordance with the NIH Guide for the Care and Use of Laboratory Animals and were approved by the Institutional Animal Care and Use Committee at Northwestern University.

#### 2.4.1 Evaluation in cats

To assess the effects of the optrodes on cochlear function after implantation, auditory brainstem responses (ABRs) to acoustic clicks and pure tone bursts were monitored before surgery and at several time points after surgery and implantation. For the procedure, the cats were sedated with Telazol (5 to 10 mg/kg, intramuscular) and were given atropine (0.04 mg/kg, subcutaneous). Vitals, such as heart rate, breathing rate, and $O_{2}$ saturation, were monitored with a Bionet BM3 vet system (Bionet America, Inc. Tustin, California, USA). Body temperature was maintained with a water-based heating blanket (T/Pump Localized Therapy system, Stryker Global Headquarters, Kalamazoo, Michigan, USA). Three needle electrodes were placed under the skin to measure ABRs by subtracting ipsilateral mastoid from vertex potentials measured relative to a ground electrode placed in the neck. The contralateral ear was blocked during testing to reduce any possible acoustical crosstalk. Acoustic stimuli were generated by a voltage command presented at a rate of 4 Hz to a Beyer DT770Pro headphone, which was calibrated with a Brüel and Kjær 1/8-in. microphone (Norcross, Georgia). The speculum of the speaker was placed directly in front of the ear canal (quasi free field). The carrier frequency of the tone bursts started at 32 kHz and was decreased by 2 steps/octave over 5 octaves. The maximum sound level at each frequency varied between 71 and 101 dB (re 20  μPa), depending on the frequency. For each frequency, the sound level was decreased stepwise by 5 dB until a visible ABR could not be seen to determine ABR thresholds. The ABR electrodes were connected to a differential amplifier (ISO-80, WPI). The amplifier has a high-input impedance ($10^{12}\Omega$) and was set to 80 dB amplification. Further amplification (10×) and bandpass filtering (0.3 to 3 kHz, −48  dB/octave) of the signal was performed by a digital filter, an IP90 (Frequency Devices, Ottawa, Illinois). The sampling rate was 250 kHz and 1024 trials were averaged for each stimulation. The threshold was defined as an ABR waveform that was visible above the noise floor of the recordings. After 1024 averages, the noise floor was typically 0.5  μV (peak-to-peak). At the conclusion of the hearing test, each animal recovered from anesthesia was returned to its home cage.

#### 2.4.2 Cochlear implantation in cats

**Fig. 4:**
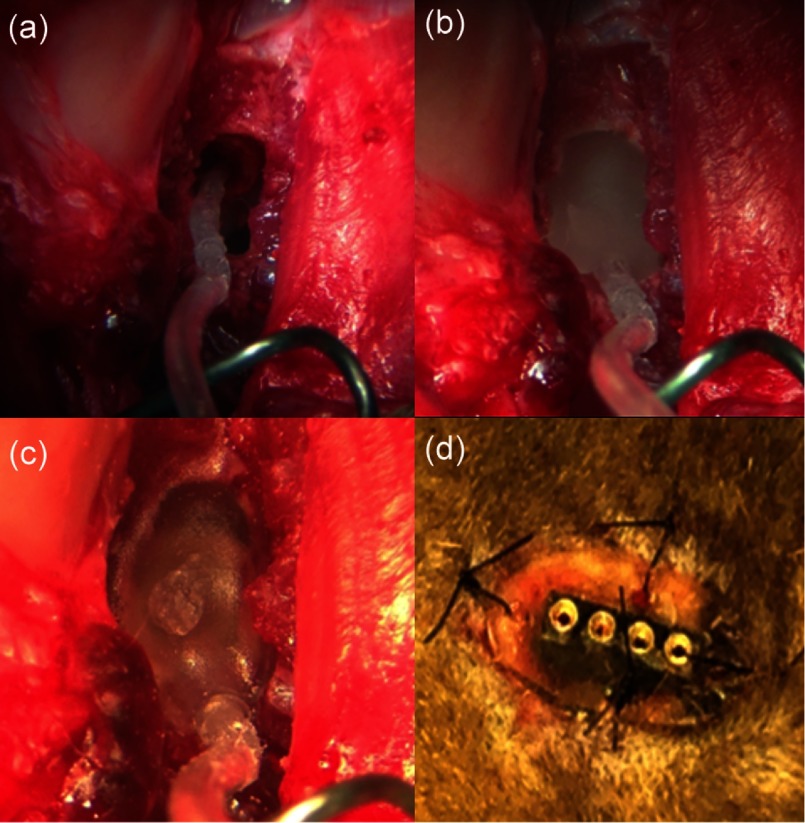
The implantation of the optrode into a cat cochlea. (a) The optrode was inserted into a cat cochlea through the cochleostomy, (b) the optrode was fixed to bulla with dental acrylic, (c) the second layer of dental acrylic, and (d) the transcutaneous connector was secured onto the lower neck skin.

In preparation for the implantation surgery, each animal was premedicated with Telazol (2 to 4 mg/kg, intramuscular), butorphanol (0.4 mg/kg, subcutaneously), and atropine (0.04 mg/kg, subcutaneously). Intravenous catheters (22G) were placed in the left and right cephalic veins, and Ringer’s solution containing 2.5% dextrose was given throughout the length of the procedure. Anesthesia was maintained with isoflurane (1% to 3%). Only the left ear was implanted with the optrode. The surgical area was aseptically prepared. A “C” shaped incision was made behind the left pinna, and the bulla was surgically accessed. An opening, $∼5\times 5\;{\mathrm{mm}}^{2}$, was created in the bulla with a motorized drill (Micro-Torque II, WPI) and a 3-mm cutting drill bit to visualize the basal turn of the cochlea. The cochleostomy was then made with a cutting drill bit (1 mm) attached to the motorized drill. The optrode was inserted about 5 mm through the bulla and the cochleostomy into scala tympani of the cochlea [Fig. 4(a)]. The optrodes were then secured at the bulla with acrylic. The acrylic not only secured the optrode but also sealed the bulla [Figs. 4(b) and 4(c)].

A small incision, 1-cm long, was made in the skin between the scapulae. The wire bundles to the optrodes were tunneled under the skin from the bulla to the scapular incision, where the electrical connector was sutured to the skin [Fig. 4(d)]. The incisions were closed in several layers with interrupted sutures. Postoperatively, the animal was monitored daily and received buprenex (0.005 to 0.01 mg/kg, subcutaneous, 2×/day for 2 to 3 days) and meloxicam (0.1 mg/kg, oral, 1×/day for 3 to 4 days) for pain management. No vestibular deficits were seen in any of the animals.

#### 2.4.3 Acute laser test in cat and guinea pig cochleae

Infrared optrodes were tested acutely in normal hearing cat and guinea pig cochlea. Guinea pig surgery has been described previously. After the cochleostomy was created with a hand-drill, a two-channel infrared optrode with high-output power VCSELs or SELDs was inserted into the animal cochlea. While inserting the optrode, care was taken such that the light-emitting window was facing the modiolus. VCSELs had a center wavelength of 1860 nm, SELDs of 1850 nm, and were operated at 100-μs pulse duration and 100-pps repetition rate. The test current levels were from 0 to 600 mA. The corresponding voltage ranged from 0 to 2 V. The maximum radiant energy was 20  μJ/pulse (measured above the optrode in air using a Coherent J-50-LP-1A energy sensor). Compound action potentials (CAPs) were recorded with an electrode placed at the round window. The recording system was the same as for the ABR recordings, except that the amplification for the CAP measurements was 60 dB.

### 2.5 Imaging at the European Synchrotron Radiation Facility

Tomographic data sets were acquired at beamline ID19 of the European Synchrotron Radiation Facility (ESRF) in Grenoble, France. Due to the 150 m-length of the beamline, excellent coherent properties of the x-ray wave fronts at the position of the experiment allow for very sensitive imaging by means of propagation-based phase contrast. In order to reduce dose to the sample tissues while maintaining a high contrast, the beamline’s single-harmonic undulator u13 (26.3 keV) was chosen. In addition, a 1-mm-thick diamond, a 2.8-mm-thick aluminum absorber, and a 0.5-mm-thick polished Beryllium exit window, the beamline was operated optics-free (pink). For detection, an in-house-developed indirect system was used, combining two commercial lenses (Hasselblad) in tandem-design. Using the ratio of the focal distance of the two lenses, here 100 and 200, the magnification was set to 2×. The lenses project the luminescence image of a 500-*μ*m-thick LuAG:Ce (Ce-doped Lu3Al5O12) single-crystal scintillator onto the sensor of a commercial camera (pco.edge, type: 5.5, 2520×2160  pixels, each 6.4-μm pixel size). At the conclusion of the experiment, the projections were used to reconstruct the samples. Custom written phase retrieval software was used for the reconstructions.^9^

### 2.6 Statistics

Average and standard deviations were calculated. Statistical analysis was completed on the ABR data to determine any significant elevation of threshold following implantation. An ANOVA was used to determine statistical significance, with the null hypothesis indicating no threshold difference between the two conditions. A one-tailed test was used for the postoperative measurement since a threshold decrease following cochlear implantation was highly unlikely.

## 3 Results

### 3.1 In Vitro Insertion of Test Electrodes

**Fig. 5:**
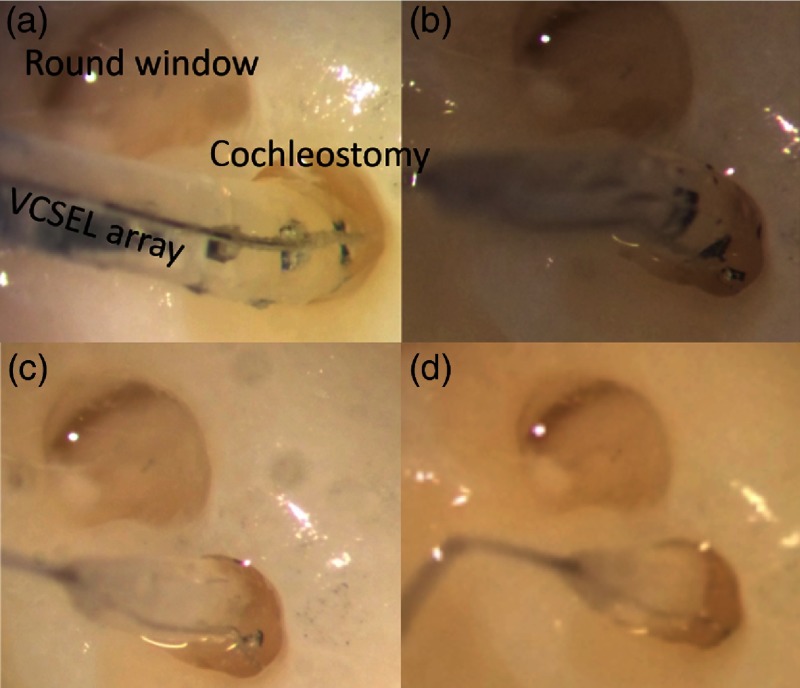
The insertion of the sham optrode into a cadaveric cat cochlea. (a) The landmarks of the magnified view of the cochlea, the round window, the cochleostomy, and the optrode. (b)–(d) The progressive insertion of the optrode into the cochlea. In panel (d), the entire optrode is inserted into scala tympani. The length of insertion is about a 6 mm. Considering the spacing of the optical sources and insertion depth, the maximum number of VCSELs that can be inserted into the cat cochlear at this time is five.

The five panels of Fig. 5 illustrate the insertion process of a sham optrode into a cadaveric cat cochlea. The sham optrode has five optical sources but the sources are only connected to the backbone. In Fig. 5(a), the electrode tip is just inserted through the cochleostomy and in Fig. 5(d), the entire optrode was inserted. After optimizing the optrode’s shape, functional ones were assembled. The longest insertion depth of an optrode in the cat cochlea was 6 mm, which was acceptable for 5 red VCSELs, or 4 infrared VCSELs or SELDs arrays, or 4 blue LEDs.

### 3.2 Insertion Force Measurements

**Fig. 6:**
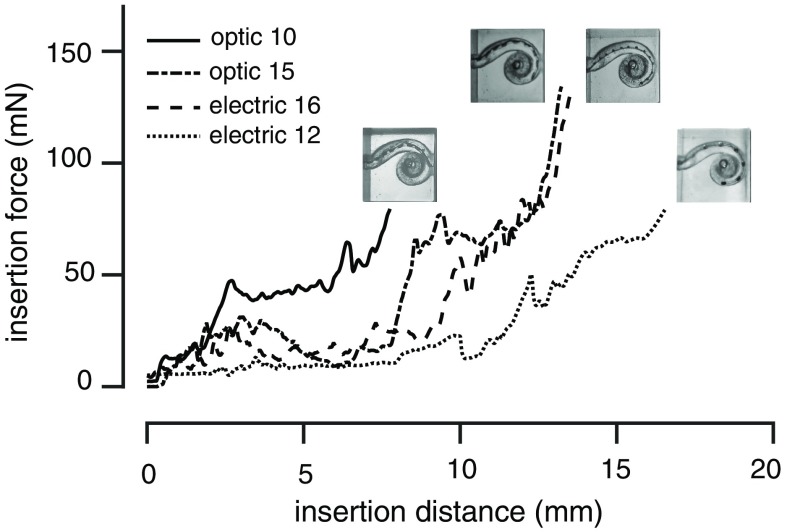
The changes in force during insertion in a model of the human scala tympani at different depths of insertion for four arrays. The optrode has 10 (blue line) or 15 (turquoise line) μLEDs. The electrical alone arrays have 16 or 12 contacts. The placement of each array in the model is shown in the four corresponding inserts.

Insertion force was measured with different custom-made optrodes in cat and human cochleae. For the short and thin arrays, the insertion forces were relatively small (<5  mN, Table 1). Insertion force was also tested with a plexiglass model of the human scala tympani, which allowed a direct view of the insertion depth (Fig. 6). Four arrays were tested in this model: two custom-made optrodes and two electrical arrays from contemporary CI systems (Table 1, Fig. 6). The insertion force and depth were comparable between the optrode with 15 contacts and the 16-channel electrode. Of note, the insertion depth was shorter than the entire length of the optrodes and electrodes. All optrodes and electrodes could be inserted with full length by hand [Figs. 2(g)–2(i)].

**Fig. 7:**
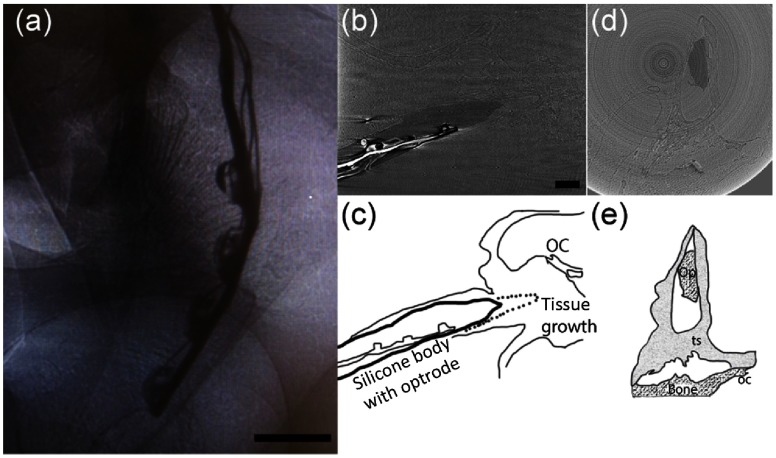
(a) An x-ray projection of an inserted optical array *in situ* in a cat cochlea. The thick wire is the backbone and acts as a heat sink. The thin wires connect to the anodes of the optical sources. The scale bar represents 500  μm. (b) and (c) The same array after the reconstruction and its sketch. The optical sources irradiate Rosenthal’s canal. A thin layer of tissue can be seen around the electrode, which has been slightly retracted (dash line in the sketch). The organ of Corti (OC) is also marked. The scale bar represents 500  μm and is used for the following panels. (d) and (e) The cross section of the same array after the reconstruction and its sketch. The optrode (Op), tissue, bone, and OC are marked in the sketch.

The placement of the optrodes in the cat cochleae was also examined by x-ray microtomography with synchrotron radiation. Figure 7(a) shows a typical projection obtained during the scans. The reconstruction of the optrode is shown in Figs. 7(b) and 7(d). Corresponding sketches are shown in Figs. 7(c) and 7(e).

### 3.3 In Vivo Functional Testing in the Guinea Pig Animal Model

**Fig. 8:**
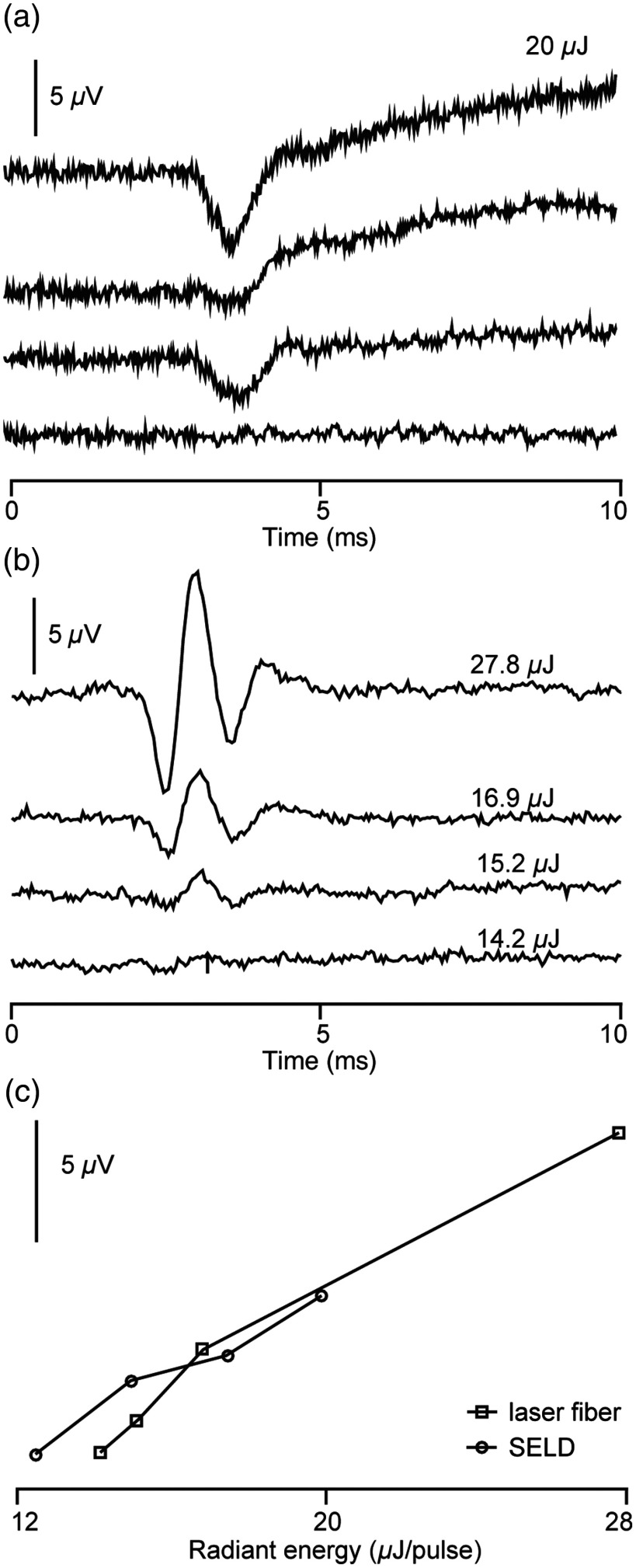
The traces show CAPs evoked with an SELD and an optical fiber in the same animal (guinea pig). (a) CAP responses evoked by a SELD operated at different current levels. The energy output measured prior to the *in vivo* test was 20  μJ/pulse with 600-mA current input. The four traces represent the current input of 600, 500, 400, and 300 mA. The traces are the averaged responses to 20 stimulus presentations. (b) CAPs evoked by delivering the radiant energy with an optical fiber at different energy levels. The traces are the averaged responses to 100 stimulus presentations. (c) The CAP amplitudes at different radiant energies for both the SELD and the optical fiber.

SELDs were assembled into arrays, which could be inserted into the guinea pig cochlea for *in vivo* functional testing. At an input current of 600 mA, the maximum radiant energy emitted from the SELDs was 15.3±4.9  μJ per pulse (n=10), ranging from 8 to 20  μJ per pulse. The energy of each SELD will be screened in future assemblies of the optrodes to eliminate the variation. The energy was measured in air with a Coherent J-50-LP-1A energy sensor. The SELDs arrays were inserted through the cochleostomy to stimulate the base of the cochlea. Optical pulses were 100  μs in duration and were delivered at 100 Hz. During the experiments, the test current levels were increased in 6 equal steps from 0 to 600 mA [Fig. 8(a)]. The amplitude of the CAPs dropped with decreasing current amplitude and reached a minimum at 300 mA [Fig. 8(a)]. CAPs also disappeared when the SELD arrays were rotated in or partially extracted from the cochleostomy so that the emitting side was no longer facing the SGNs.

CAP amplitudes in response to INS, which were obtained with the SELDs, were compared to CAP amplitudes measured in the same animal by delivering the radiation with an optical fiber [Figs. 8(b) and 8(c)] coupled to a table-top laser (Lockheed Martin Aculight Corp., Bothell, Washington).

### 3.4 In Vivo Test in Cats

For the cochlear stimulation devices, it is important that they are both biocompatible and can evoke neural responses. In cat cochleae, the power of the VCSEL array was too low to evoke measurable ABRs. The results of the chronic experiments demonstrate that the optrodes are biocompatible and that chronic implantation does not damage cochlear function over time. Functional tests with more powerful SELDs were done in the guinea pigs as shown previously.

**Fig. 9:**
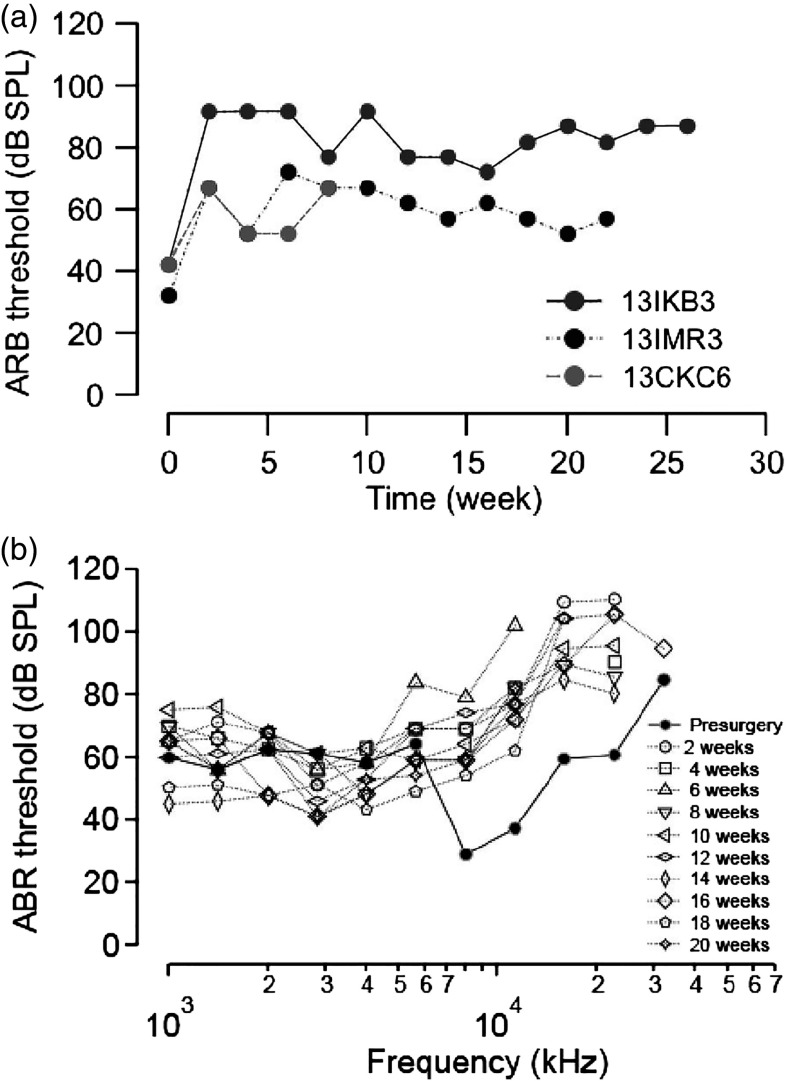
The ABR thresholds to acoustic stimuli pre- and postimplantation. (a) Click evoked ABR thresholds at different time points in three cats. Click thresholds were elevated after optrode implantation, but then remained consistent in the months after; (b) pure tone evoked ABR thresholds at different times after implantation for animal 13IMR3. The optrode implantation caused high-frequency hearing loss (at 32 kHz) and elevated thresholds among 8 to 22.6 kHz, but little change was noted below 8 kHz.

After the *in vitro* long-term testing, the optrodes were implanted into the left cochleae of a cat. Figures 4 and 5 show the optrode insertion and the transcutaneous connector to the current source. The current sources were small computer controllable laser diode drivers developed by Lockheed Martin Aculight (LMA), which fit into the backpack of a cat. Alternatively, a commercially available high-power precision source, LDX-32400 (ILX Lightware, Bozeman, Montana), was used. Optically and acoustically evoked ABRs were recorded before implantation (baseline) and two weeks after the surgery (Fig. 9). Thereafter, acoustically evoked ABRs were measured every two weeks up to 26 weeks after the surgery. Note that the cat study aimed to determine the biocompatibility of the optrodes and to determine whether implantation and materials will lead to a deterioration of cochlear function over time. Cat number 13IKB3 was implanted with red VCSELs. Cats 13IMR3 and 13CKC6 were implanted with the low-power infrared VCSELs. 13IKB3 was excluded from measuring the optical ABRs (oABRs). No oABRs were evoked by the low-power VCSELs. In pulsed operation mode, the highest output power of low-power VCSELs is about 70 mW (7  μJ/pulse), which is about the radiant energy required to reach stimulation threshold as determined in previous experiments.^18^^,^^32^ Sound levels to evoke an ABR with acoustic clicks were elevated immediately after surgery by about 50, 35, and 25 dB in cat 13IKB3, 13IMR3, and 13CKC6, respectively. Thresholds were determined two weeks after the surgery. No further changes in threshold were observed after the placement of the optrode [Fig. 9(a)]. Threshold elevations occurred mostly at frequencies above 22 kHz, where the optrode was located [Fig. 9(b)]. No acoustic responses could be recorded for stimulation frequencies above 22 kHz in all three cats, and sound levels to reach the threshold for an ABR were elevated at frequencies between 8 and 22 kHz by 25 dB in cat 13IMR3.

## 4 Discussion

### 4.1 Requirements for the LDS

#### 4.1.1 Physical design of the light delivery system

In the ideal scenario, the CI electrode design would be tailored to each patient. This would allow the optimal placement and orientation of the optical sources toward the auditory neurons. Since this technology has not matured yet, the electrode array was fabricated in a circular form to best fit the different area configurations of scala tympani. Ample data are available in the literature describing the dimensions of scala tympani of the human cochlea.^81^^–^^84^ The dimensions of the LDS should taper from 1 to 0.47 mm. The length of the electrode/optrode should be shorter than 27 mm.

#### 4.1.2 Energy requirements

In a recent paper, we reported the radiant energy required for INS in guinea pigs and cats.^32^ At the tip of the optical fiber, it was on average 14.1±8.1  μJ/pulse for single units in the central nucleus of the inferior colliculus (ICC) and 17.2±13.9  μJ/pulse for CAPs. The variation of the radiant energy at the tip of the optical fiber to reach stimulation threshold was large with a range of 4.8 to 47  μJ/pulse. After correcting for the distance between the tip of the optical fiber and the modiolus, the radiant energy on target was between 1.4 and 16.4  μJ/pulse, on average 4.1±1.9  μJ/pulse for ICC single units and 7.2±4.7  μJ/pulse for CAP responses. The radiant energy of the SELDs emitting light at 1850 nm was typically in the range between 8 and 20  μJ/pulse. This reaches the threshold for stimulation in most of the cases. It is also below the threshold for which cochlear damage was detected.^31^^,^^85^ It is important to explore and implement methods to reduce the amount of energy required for stimulation. One possibility is combined optical and electrical stimulation. Duke et al.^86^^,^^87^ have demonstrated that subthreshold electrical stimulation can lower the threshold for INS by a factor of about two. More recently, we have shown in deaf white cats that combined optical and electrical stimulation reduces the threshold for INS in the cochlea.^88^

### 4.2 Enabling Technology

#### 4.2.1 Optical fibers and fiber bundles

A detailed study on the design of an LDS using optical fibers was completed previously.^73^ To accommodate the optical fiber bundle in scala tympani of a human inner ear, the maximum diameter of the optical fiber bundle must be <0.7  mm. In their experiments, the authors inserted single silicone-coated fibers or small silicone-coated fiber bundles with core/cladding diameters of 20/25, 50/55, 50/125, and 105/125  μm. The results showed that thicker fibers (50/125 or 105/125) broke after being inserted about 10 mm. At this insertion depth, the tip of the electrode reaches a site along the cochlea where a steep turn occurs. Thin optical fibers and their corresponding optical fiber bundles up to eight fibers could be inserted up to 20 mm into scala tympani of a cadaveric human cochlea. The results also demonstrate that optical fibers pose a challenge if more than eight channels are required. Moreover, it is not clear whether the maximal energy delivered through those fibers is sufficient for stimulation. Based on their experience and working with glass optical fiber, we have not attempted to build a multichannel optical implant to be deeply inserted into a cochlea.

#### 4.2.2 Bundles of waveguides

Waveguides are typically made out of dielectric materials. Their elastic modulus is about 3.2 GPa versus ∼70  GPa for fused silica. Depending on the geometry of the fiber’s cross section, waveguides are about 20 to 30 times more compliant than optical fibers made of fused silica. Waveguides core structure has a high index of refraction and is surrounded by a material with lower permittivity, the cladding. The structure guides optical waves by total internal reflection. Consequently, the extinction coefficient for the core material must be low for the radiation wavelength. Although waveguides are readily available for the visible range, they are not for the near-infrared or infrared. According to the literature, materials exist that are suitable for insertion into the cochlea and have good transmission at the wavelength of interest, λ=1840 to 2100 nm.^89^^–^^92^ Examples are polyimides, such as Kapton^®^. It is commercially available through DuPont. The index of refraction of the material at λ=1860  nm is about 1.6. At those wavelengths, Kapton^®^ waveguides (size 10  μm) have propagation losses around 1 dB/cm. Fluorinated polyimides are even better at transmitting the radiation. They have propagation losses of about 0.6 dB/cm.^90^ The cladding of the waveguides could be Teflon or Silastic^®^. The latter is used to encapsulate the waveguides. Silastic^®^ and Teflon have an index of refraction of about 1.3 at λ=1860  nm. This gives a critical angle of ∼60  deg.

#### 4.2.3 Optogenetics

For the mouse, it has been demonstrated that the blue light radiation at $2.2\;\mu J/{\mathrm{mm}}^{2}$ evokes an auditory response. This is about 7 to 70 times less than the energy required for INS in the gerbil or cat. Existing μLEDS are powerful enough for stimulation. The technology has advanced to produce miniature sources^93^^–^^95^ that can be inserted in cochleae of small animals. However, challenges are expected for larger distances from the light sources and bone lies in the beam path. As indicated before, for radiation wavelengths up to about 1064 nm, the extinction coefficient for the radiation is governed by the scattering of the photons. For wavelengths above 1064 nm, the absorption of the photons by the fluids in the tissue becomes the dominant factor. In other words, bone in the beam path will drastically reduce the radiant energy and will widen the beam path and consequently will affect the selectivity of optical stimulation.

## 5 Conclusion

With our work, we have demonstrated that LDSs can be fabricated for optical stimulation with INS. With the current design, optrodes can be inserted into a human scala tympani model up to ∼360  deg. Optrodes made with SELDs were able to evoke auditory responses in guinea pigs. Chronic implantation of the optrodes did not elevate acoustically evoked ABRs over 26 weeks in cats. Future studies will focus on developing optrodes with SELDs and testing functionality in longitudinal studies.
